# Chemical Composition and Cosmeceutical Potential of the Essential Oil of *Oncosiphon suffruticosum* (L.) Källersjö

**DOI:** 10.3390/plants10071315

**Published:** 2021-06-28

**Authors:** Selena O. Adewinogo, Rajan Sharma, Charlene W. J. Africa, Jeanine L. Marnewick, Ahmed A. Hussein

**Affiliations:** 1 Chemistry Department, Bellville Campus, Cape Peninsula University of Technology, Symphony Road, Bellville 7535, South Africa; selenaorangoeunice@gmail.com (S.O.A.); sharmar@cput.ac.za (R.S.); 2 Department of Medical Biosciences, University of the Western Cape, Bellville 7535, South Africa; cafrica@uwc.ac.za; 3 Applied Microbial and Health Biotechnology Institute, Cape Peninsula University of Technology, Symphony Rd., Bellville 7535, South Africa; marnewickj@cput.ac.za

**Keywords:** essential oils, *Oncosiphon suffruticosum*, antioxidant, antibacterial, tyrosinase inhibition, sun protection factor

## Abstract

The South African medicinal plant *Oncosiphon suffruticosum* (L.) Källersjö is an important remedy used to treat chronic, respiratory, and skin ailments. From the essential oil (EO) extracted by the hydrodistillation, sixteen constituent components were identified with oxygenated monoterpenes: camphor (31.21%), filifolone (13.98%), chrysanthenone (8.72%), 1,8-cineole (7.85%), and terpinen-4-ol (7.39%) as predominant constituents. In the antibacterial activity study, the EO was found most susceptible against *Pseudomonas aeruginosa* with an MIC of 6.4 mg/mL; however, it showed the same activity against *Staphylococcus aureus* and *Escherichia coli* with an MIC value of 12.8 mg/mL. The sun protecting factor (SPF) of the EO was found to be 2.299 and thus establishing it as a potentially important cosmeceutical for sunscreen applications. This is the first report investigating the essential oil of *O. suffruticosum* for its chemical composition and skin-related in vitro biological activities viz antibacterial, antioxidant capacity, antityrosinase, and sun protection factor.

## 1. Introduction

Essential oils (EOs) are aromatic oily liquids composed of a complex mixture of volatile compounds and are produced by aromatic plants as secondary metabolites. The volatile constituents of EOs have been important materials for preventing and treating human diseases since the early days [1]. Although mainly used for their agreeable scents, EOs present themselves as excellent candidates to meet the current beauty industry’s demands for two principal reasons. Firstly, research backs up their efficacy as valuable cosmeceuticals. They have been shown to exhibit properties of antimicrobials [2,3], antioxidant agents [4,5], antityrosinase agents [6,7,8], sunscreens [9,10], natural preservatives [11], natural sources of fragrance [12], as well as inhibitors of skin’s degradation enzymes (collagenase and elastase) [13]. Secondly, the small lipophilic molecules that make up their composition grant easy penetration through the skin layers [14].

South Africa (SA) is home to an important and rich botanical diversity. The country boasts over 30,000 flowering species with high endemism and is ranked third in biodiversity in the world [15,16]. A significant fraction of aromatic plant species contributes to this rich heritage. To date, oil-rich plant species recorded in South Africa belong to the Asteraceae family (2300 species), Rutaceae family (290 species), and Lamiaceae (235 species) family [16].

*Oncosiphon* Källersjö is an aromatic genus of the Asteraceae family and Anthemideae tribe that counts seven species. Some species of the genus were formerly classified in the *Pentzia* Thunb. and others in *Matricaria* L. genera. However, the *Oncosiphon* genus later arose due to the morphological differences recorded in the now-*Oncosiphon* species which were not present in the *Pentzia* genus. Most of the *Oncosiphon* species are native to the Greater Cape Floristic Region except for *O. piluliferum* (L.f.) Källersjö and *O. suffruticosus* (L.) Källersjö. These two species also grow in Australia and are respectively known as Globe Chamomile and Calomba Daisy. *Oncosiphon* species bear the Afrikaans name “stinkruid” which means stinkweed due to their pungent aroma. Among them, *O. piluliferum*, *O. suffruticosus*, and *O. africanum* are important materials of Cape Dutch ethnobotany and Khoi-San medicine [17].

**Figure 1:**
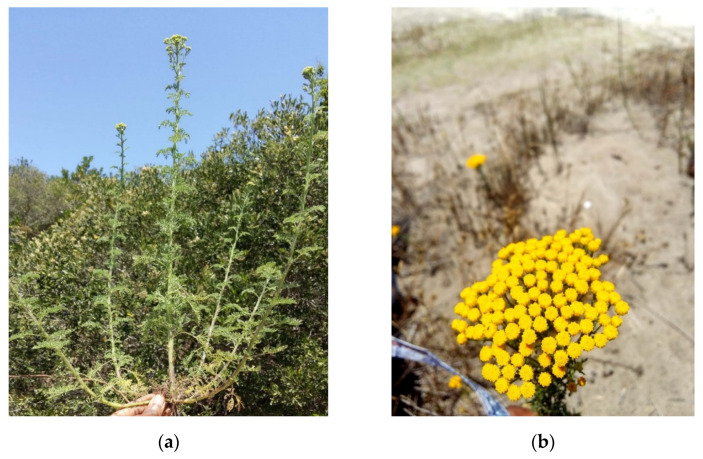
Photographs of *O. suffruticosum*. (**a**) Uprooted branch; (**b**) flower heads. These photographs were taken during the summer season (December, 2018) at the Cape Flats regions of Cape Town, South Africa.

The *O. suffruticosum* (L.) Källersjö herb features hairless and thin leaves (Figure 1). It bears a typical sharp and powerful scent like other *Oncosiphon* species. The herb grows up to 50 cm tall annually and is distributed in the southern part of Africa from the Western Cape to Namibia [18]. In traditional healing practices, oral administrations aim to treat asthma, gastric disorders, convulsions, diabetes, rheumatic fever, typhoid fever, colds, and influenza [19,20]. Additionally, the herb is used topically as a leaf poultice to treat scorpion stings and inflammation [20].

Since time immemorial, plants have been renowned sources of bioactive materials used in traditional therapies and a reservoir for innovative cures in modern medicine. The use of plants ranges from culinary preparations, medicine, to perfume compositions [21]. However, only a few SA medicinal plants are explored commercially [22] and investigated scientifically [20]. According to the literature, the essential oil of *O. suffruticosum* has never been studied before. In the quest to explore the South African flora for novel cosmeceutical ingredients, the aim of the present research was to elucidate the chemical composition and study the biological studies, antimicrobial activity, antioxidant capacity, antityrosinase activity, and photoprotection of the essential oil of *O. suffruticosum*.

## 2. Results and Discussion

### 2.1. Chemical Composition of O. suffruticosum Essential Oil

The hydrodistillation of fresh aerial parts of *O. suffruticosum* gave an average essential oil yield of 0.23% (*v/w*). According to the present GC-MS analysis, sixteen components representing 85.09% of the EO in composition were identified (Table 1).

**Figure 2:**
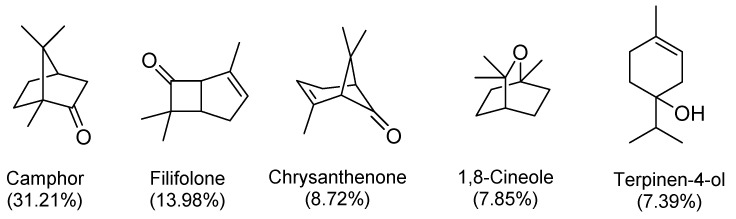
Major components detected in *O. suffruticosum* essential oil.

The major constituents of the EO were found to be the hydrocarbons and oxygenated monoterpenes amounting to 84.64%, of which the oxygenated monoterpenes were dominant by 76.49%. The only identified sesquiterpene was found to be caryophyllene oxide present as 0.45%. No hydrocarbon sesquiterpenes were detected. The major constituents were found to be oxygenated monoterpenes: camphor (31.21%), filifolone (13.98%), chrysanthenone (8.72%), 1,8-cineole (7.85%), and terpinen-4-ol (7.39%) (Figure 2).

As per the literature, the *O. suffruticosum* essential oil had never been studied before as it is for other plants of the same genus. However, according to the results obtained, a chemical link to its historical classification in the *Pentzia* genus was observed. Like *O. suffruticosum* EO, the chromatographed EOs of *Pentzia incana* [26] and *Pentzia punctata* [27] have shown to possess a significant content of camphor of up to 47.9% and 27.3%, respectively. Additionally, 1,8-cineole was also found as a major compound in *Pentzia incana* with up to 16.7% [26].

### 2.2. Antibacterial Activity: Minimum Inhibitory Concentration (MIC) Using the Broth Microdilution Method

The evaluation of the cutaneous antibacterial effect of *O. suffruticosum* essential oil was assessed against three bacterial strains, *Staphylococcus aureus*, *Pseudomonas aeruginosa*, and *Escherichia coli*, in the broth microdilution susceptibility assay. The results were taken as the lowest concentration inhibiting visible bacterial growth as detected by the *p*-iodonitrotetrazolium chloride (INT) reagent and expressed in mg/mL as presented in Table 2.

The MIC of *O. suffruticosum* EO was detected as 12.8 mg/mL for *S. aureus* and *E. coli*, whereas it was found twice as lower for *P. aeruginosa* and detected as 6.4 mg/mL. According to Van Vuuren [16], essential oils with an MIC ≤ 2 mg/mL can be taken as effective. Therefore, according to these results, *O. suffruticosum* EO may be classified to possess low to moderate antibacterial activity. These findings correlate well with the chemical composition of this EO. Indeed, it is known that the chemical structure of terpenoids parallels their activity [28], whereby the presence of an oxygen function in the framework enhances their antimicrobial properties [29]. The phenol and aldehydes are often characterized by the highest antibacterial activity [30] followed by the alcohols which are usually bactericidal rather than bacteriostatic, then the ketones and the terpene hydrocarbons which have weak activities [29]. In the EO of *O. suffruticosum*, phenols were not detected and only one aldehyde terpene was detected, 2-ethylidene-6-methyl-3,5-heptadienal, as 5.71%. The predominant functional moieties were ketones, alcohols, and terpene hydrocarbons by 78.93% which could explain the lower bacterial inhibitory activity.

### 2.3. Antioxidant Capacities

Free radicals chain reactions culminate in oxidative stress when the number of free radicals surpasses the number of systemic defenses, the antioxidants, in the target cell [31]. Oxidative stress in the skin is expressed by blotchy pigmentation, sagging skin, and wrinkles [32]. The strength of the antioxidative potential of *O. suffruticosum* essential oil was evaluated by four in vitro antioxidant capacity assays. The selection of the assays considered covering electron transfer (ET)- and hydrogen atom transfer (HAT)-based mechanisms. The ET-based methods selected were the 2,2-diphenyl-1-picrylhydrazyl (DPPH), 2,2′-Azino-bis(3-ethylbenzothiazoline-6-sulfonic acid) (ABTS), and Ferric reducing antioxidant power (FRAP) assays although DPPH and ABTS can involve both HAT and ET mechanisms [33,34]. The HAT-based method selected was Oxygen radical absorbance capacity (ORAC) assay [35,36]. The results are summarized in Table 3.

In the DPPH assay, the values of % radical scavenging activity (% RSA) of the essential oils were found to be extremely poor, 10.03 ± 1.02%, 8.38 ± 0.24%, and 7.06 ± 0.20% at 2, 1, and 0.5 mg/mL, respectively. Additionally, they were significantly lower than that of Trolox^®^ positive control found as 94.94 ± 0.02%, 94.78 ± 0.06%, and 94.45 ± 0.04% at the respective concentrations tested. In the ABTS assay, the % RSA’s were found to be higher and comparable to the gallic acid positive control. The values were found to be 87.17 ± 0.76% to 71.46 ± 0.04% for the EO vs. 97.97 ± 0.13% to 98.05 ± 0.03% for the positive control in the 2 to 0.5 mg/mL concentration range. The higher performance of the EO in the ABTS assay was expected as ABTS•+ are more reactive than DPPH radicals [34]. However, the difference in antioxidant strength between the EO and gallic acid was evident in the discrepancy in Trolox^®^ equivalent values which were 100-fold higher for gallic acid than those of the EO. Moreover, the EO was found to be −505.8 ± 80.8 μmol (AAE)/L at 2 mg/mL in the FRAP assay against being 635,500 ± 4070.9 μmol AAE/L for gallic acid positive control, and 6701.8 ± 57.2 μmol TE/L in the ORAC assay at the same concentration against 26,904 ± 328.2 μmol TE/L for EGCG positive control. The antioxidant capacity of a substance assesses the amount of antioxidant which reacts with the oxidant [37]. Overall, the EO was found to exhibit a much weaker performance than the reference controls. Therefore, the results indicated that the EO possesses poor to moderate antioxidant capacity.

### 2.4. Tyrosinase Inhibition

Tyrosinase (EC 1.14.18.1), also known as polyphenol oxidase, is a copper-containing enzyme that has a central role in the production of melanin, the pigment responsible for the color of the skin. It catalyzes the first two steps of the multiphase process of melanogenesis, the biosynthesis of melanin. Today, tyrosinase inhibitors are increasingly prevalent cosmeceuticals’ ingredients aiming to treat hyperpigmentation problems caused by the overproduction of melanin in the skin [38]. In the present work, the *O. suffruticosum* essential oil was tested in the tyrosinase inhibition assay exploring the monophenolase activity of the enzyme by monitoring the absorbance of L-DOPA (λ_490_ nm) using L-tyrosine as a substrate. The essential oils were tested at 200 μg/mL and 50 μg/mL and compared to kojic acid, a standard tyrosinase inhibitor used in cosmetics, at the same concentrations. The results were obtained as presented in Table 4.

*O. suffruticosum* EO exhibited significantly lower tyrosinase inhibition values than kojic acid at both concentrations tested. At 200 μg/mL, the EO was found to be 61.46 ± 11.00% against 96.24 ± 3.62% for kojic acid and at 50 μg/mL, the EO was found to be 26.14 ± 3.74% against 98.34 ± 0.80% for kojic acid. These values indicate that the enzyme inhibition is concentration dependent, and *O. suffruticosum* EO is a relatively weak tyrosinase inhibitor.

### 2.5. Sun Protection Factor (SPF)

Solar UV rays are recognized as the main contributor to extrinsic cutaneous aging in humans [39,40,41]. Chronic exposure to ultraviolet radiation (UVR) induces various dermatological problems including skin cancer [42]. Herein, the SPF of *O. suffruticosum* essential oil was determined by measuring the absorbance of a dilute hydroalcoholic solution of EO (0.1% *v*/*v*) at 290–320 nm at 5 nm interval then calculated using the equation given by Mansur et al. [43]. The results are presented in Table 5.

According to the study, the essential oil of *O. suffruticosum* was found to possess an SPF value of 2.299. It has been reported that an SPF value above 2 is noteworthy [45,46]. Such a substance may block UV radiation by approximately 57% [45,46,47]. Therefore, the results establish *O. suffruticosum* EO as an important cosmeceutical for sunscreen formulation.

In an attempt to compare the biological activities of the plants which are rich in the major components found in the *O. suffruticosum* EO, the essential oils of *Cinnamomum camphora*, *Artemisia herba-alba*, *Eucalyptus globulus*, and *Melaleuca alternifolia* were selected as representative examples with camphor, chrysanthenone, 1,8-cineole, and terpinen-4-ol as respective major components. The results from the literature search indicated that mainly these essential oils have been studied for their antibacterial and antioxidant properties and they showed variable degree of activities.

The *C. camphora* essential oil contains camphor as the main constituent. The sample collected from Pantnagar, India, was effective against *Pasturella multocida* but not against *Salmonella enterica enterica* and *Escherichia coli* [48]. *C. camphora* oil from Nepal also showed marginal activity against *B. cereus* and *S. aureus*, with a MIC = 313 μg/mL [49]. During an antioxidant study by the DPPH assay, the IC_50_ value of the *C*. *camphora* essential oil was found to be 31.85 µL/mL, whereas that for the reference butylated hydroxytoluene (BHT) was reported to be 7.6 µg/mL [50].

Essential oil of *A. herba-alba* from Makther Seliana, Tunisia having camphor and chrysanthenone as major components displayed MIC (µg/mL) values of 100, 50, and >100 against *S. aureus*, *E. coli*, and *P. aeruginosa*, respectively [51]. The antioxidant activity of *A. herba-alba* EO by DPPH assay showed an IC_50_ of 2.66 µg/mL whereas that for the synthetic antioxidant butylated hydroxyanisole (BHA) was 1.66 µg/mL [52]. At a concentration of 1 mg/mL, the *A. herba-alba* EO exhibited a tyrosinase inhibition of 31.35%, which was much lower than that of the standard inhibitor kojic acid (87.54% at 0.05 mg/mL) [53].

EOs of *E. globulus* collected from Skoura, Morocco, presented excellent activity on *E. coli* in the agar disc diffusion assay with inhibition zone diameter (izd) = 48.15 mm compared to *S. aureus* (izd = 13.5 mm) and *S. intermedius* (izd = 10.26). The MIC for *E. coli* corresponded to 0.15 mg/mL while for *S. aureus* and *S. intermedius* the values corresponded to 0.75 mg/mL and 1.08 mg/mL, respectively [54]. The main component of *E. globulus* EOs is 1,8-cineole and it has been demonstrated that this compound has antimicrobial activity against several microorganisms including *S. aureus* and *E. coli* [55]. In an antioxidant study of this plant with the DPPH method, its methanolic extract exhibited the strongest free radical-scavenging activity with an IC_50_ value of 23 µg/mL, followed by the ethyl acetate extract (IC_50_ = 29 µg/mL) and hexane extract (IC_50_ = 65 µg/mL). However, the essential oil did not show any noticeable activity with the DPPH method [56]. This activity may be attributed to the high content of phenolic compounds (542.42 mg GAE/g) in methanol extract from *E. globulus*.

Essential oil from *M*. *alternifolia* is referred to as tea tree oil, the major component of which is terpinen-4-ol present at least 30% of total oil [57]. *M. alternifolia* essential oil obtained from a commercial source in Germany inhibited the growth of *S. aureus*, *E. coli,* and *P.* *aeruginosa* at a concentration of 5% *w*/*v* [58]. An antioxidant activity study by the DPPH method indicated that *M. alternifolia* EO at a concentration of 10 µL/mL produced 80% free radical scavenging activity which was equivalent to that of 30 mM BHT [59].

No tyrosinase inhibition studies are reported for essential oil of these plants except *A. herba-alba*. There was also no report in the literature regarding the SPF studies of the essential oils of these plants. As per the above discussed results from the literature, no direct correlation could be ascertained among the biological activity and the major component of the essential oil, suggesting that the biological activity of the essential oils is because of the synergism among the components of the essential oil rather than any one of the major constituents.

## 3. Materials and Methods

### 3.1. Plant Material

The plant material (3.0 kg) was wildly harvested from the Cape Flats Nature Reserve of the University of the Western Cape in December 2018. A voucher specimen was authenticated by Hlokane Mabela and deposited at the Horticultural Sciences Department of the Cape Peninsula University of Technology.

### 3.2. Extraction of Essential Oil

The fresh aerial parts (leaves, stems, and flowers) of *O. suffruticosum* were subjected to hydrodistillation using the Clevenger-type apparatus for 3 h as per the European Pharmacopeia’s guidelines [60]. The essential oil was recovered by decantation in glass vials and stored in the dark at 4 °C until further use. The oil yield was expressed as the average percentage of volume in mL per weight in g (% *v/w*) of triplicate analyses.

### 3.3. Gas Chromatography-Mass Spectrometry (GC-MS) Analysis

The GC-MS analyses were carried out according to the in-house method and the procedure previously reported by Kuiate et al. [61] with some adjustments. The instrument consisted of an Agilent GC-7820A fitted with an HP-5MS fused silica column (30 m × 0.25 mm i.d. × 0.25 μm film thickness) and coupled with an Agilent 5977E mass selective compartment (Agilent Technologies, Inc., Santa Clara, CA, USA). The oven temperature was programmed at 50 °C for 5 min, 50–220 °C at a rate of 2 °C.min^−1^ then 220 °C temperature hold for 5 min for the first ramp. For the second ramp, the temperature was set to 300 °C at a rate of 25 °C.min^−1^. Helium was used as a carrier gas at 1 mL.min^−1^ flowrate and pressure of 7.6522 psi. Sample injection of 1 μL of 1% (*v*/*v*) solution diluted in n-hexane was splitless and operated at 250 °C. A reference standard of homologous n-paraffin series of C8-C20 (Sigma-Aldrich^®^, St. Louis, MO, USA, Cat no. 04070) was prepared and co-injected under identical experimental conditions as the samples for the determination of retention indices (RIs). The MS spectra were obtained on electron impact at 70 eV scanning from 30.0 to 650 *m*/*z*.

The identification of the constituents was achieved by computerized matching (MassHunter software, Agilent Technologies, Inc., Santa Clara, CA, USA) of each mass spectrum generated with authentic samples (if available) and with those stored in the instrument’s built-in mass spectral libraries (National Institute of Standards and Technologies, NIST), comparing of the experimental RIs [62] with those of the NIST online data collection [25] and literature [23,24]. The relative amounts of individual constituents were calculated automatically based on the total ion count detected by the GC-MS and expressed as percentage composition.

### 3.4. Antibacterial Assay

#### 3.4.1. Micro-Organisms

The essential oil was tested against three skin pathogenic bacterial strains obtained from the Medical Bioscience Department at the University of the Western Cape. These were one gram-positive strain, wild-type (WT) *S. aureus,* and two gram-negative strains, wild-type (WT) *E. coli* and wild-type (WT) *P. aeruginosa*.

#### 3.4.2. Preparation of the Media

The bacterial species were resuscitated by inoculation into Brain heart infusion (BHI) broth (Oxoid UK, Cat. no. CM1135) and incubated at 37 °C for 24 h after which, each strain was streaked aseptically onto Tryptone soya agar for single colony formation and incubated at 37 °C for 24 h. The cell suspensions were performed in sterile saline, standardized at 0.5 McFarland standard (Remel™, Kansas, Cat. no. R20410) at 1.5 × 10^8^ colony forming units (CFU)/mL. Then, the working suspensions were obtained by a second 1:100 dilution onto BHI to approximately 10^6^ CFU/mL.

#### 3.4.3. Broth Microdilution Susceptibility Assay

The broth microdilution test was performed as previously described by Lourens et al. [63] and Sartoratto et al. [64] with slight adjustments. An EO stock solution of 51.2 mg/mL was prepared with a BHI:dimethyl sulphoxide (DMSO) (1:1) solution. In a 96-well plate, 100 μL of BHI was added to the experimental wells in triplicate except in well 1. Then, 200 μL of EO stock solution was added to well 1, from which a serial dilution was performed to the last experimental well. Subsequently, 100 μL of cell suspension was added to establish the two-fold 25.6–0.2 mg/mL sample concentration range and a bacterial cell suspension of approximately 5 × 10^5^ CFU/mL. The plate was incubated at 37 °C for 20 h. After incubation, the antimicrobial activity was detected by adding 40 μL of 0.2 mg/mL INT (Sigma-Aldrich^®^, Cat no. I10406) aqueous solution. The plates were incubated at 37 °C for 1 h. The MICs were defined as the lowest concentration of essential oil that inhibited visible growth, as indicated by the color change of INT. Ampicillin (Sigma-Aldrich^®^, Cat no. A9393) was used as a positive control.

### 3.5. Antioxidant Capacity Assays

The antioxidant capacity of the *O. suffruticosum* EO was studied by the following four antioxidant assays to cover both HAT and ET mechanisms.

#### 3.5.1. 2,2-Diphenyl-1-Picrylhydrazyl (DPPH) Assay

The DPPH assay was performed according to the method previously described by Bondet et al. [65] with slight modifications. In a clear 96-well plate, 275 μL of DPPH reagent (Sigma-Aldrich^®^, Cat no. D9132) (absorbance of 2.0 ± 0.1 at 517 nm) was added to 25 μL of EO sample and Trolox^®^ (Sigma-Aldrich^®^, Cat no. 238831) positive control (2.0, 1.0, and 0.5 mg/mL). For the blank, ethanol was added instead of the sample. The total volume of the assay was 300 μL. The absorbance was read at 517 nm and 37 °C at the 6 min time point. The EO/Trolox^®^ sample was read in triplicate (n = 3). The percentage radical scavenging activity (% RSA) of the samples was calculated using Equation (1).

(1) $$\%\;RSA_{6\;min}=1-\frac{Abs_{sample}\;}{Abs_{blank}},$$

where *Abs_sample_* is the absorbance signal of the EO sample and *Abs_blank_* is the absorbance signal of the DPPH solution (ethanol in place of the sample) at 517 nm after 6 min. The results were expressed as the mean percentage of triplicate measurements (±standard deviation, SD).

#### 3.5.2. 2,2′-Azino-bis(3-Ethylbenzothiazoline-6-Sulfonic Acid) (ABTS) Assay

The ABTS assay was performed according to Re et al. [66] with slight modifications. The ABTS radical cation (ABTS•+) (Sigma-Aldrich^®^, Cat no. A1888) stock reagent was produced by reacting 5 mL of freshly prepared 7 mM ABTS solution with 88 μL of a freshly prepared 140 μM K_2_S_2_O_8_ (Merck, Cat no. 105091) then allowing the mixture to sit overnight for 16 h in the dark at room temperature. In a clear 96-well plate, 275 μL of ABTS•+ reagent (absorbance of 2.0 ± 0.1 at 734 nm) was added to 25 μL of each ethanolic Trolox^®^ working standard (50 μM, 100 μM, 150 μM, 250 μM, and 500 μM) and EO sample (2.0, 1.0, and 0.5 mg/mL). Gallic acid (Sigma-Aldrich^®^, Cat no. G7384) was used as a positive control. For the blank, ethanol was added instead of the sample. The total volume of the assay was 300 μL. The absorbance was read at 734 nm and 37 °C at the 6 min time point. The EO sample, working standard, and gallic acid sample were read in triplicate (n = 3). The percentage of radical scavenging activity (% RSA) of each EO or positive control working solution was calculated using Equation (1), where Abs_sample_ is the absorbance signal of the EO sample/positive control and Abs_blank_ is the absorbance signal of the ABTS•+ solution (ethanol in place of the sample) at 734 nm. The results were expressed as the mean percentage of triplicate measurements (±standard deviation, SD). The Trolox^®^ equivalent capacity assay (TEAC) values were reduced from the linear regression (R^2^ = 0.9980) of Trolox^®^ concentrations (μM) and the absorbance readings at 734 nm at 6 min and expressed as mean (±SD) of triplicate measurements in μmol Trolox^®^ equivalents per liter of the sample tested (μmol TE/L).

#### 3.5.3. Oxygen Radical Absorbance Capacity (ORAC) Assay

The ORAC assay was performed according to the method described by Prior et al. [67] with slight modifications. In a black 96-well plate, 12 µL of the Trolox^®^ working solutions (83 µM, 167 µM, 250 µM, 333 µM, and 417 µM prepared with phosphate buffer at pH 7.4) and EO sample (2.0 mg/mL) were added in triplicate (n = 3). Subsequently, 138 µL of fluorescein solution was added followed by 50 µL of freshly prepared by dissolving 2,2’-Azobis (2-methylpropionamidine) dihydrochloride (AAPH) (Sigma-Aldrich^®^, Cat no. 440914) in phosphate buffer (150 mg of AAPH in 6 mL buffer). (-)-Epigallocatechin gallate (EGCG) (Sigma-Aldrich^®^, Cat no. E4143) was used as a positive control. For the blank, the phosphate buffer was added in place of the sample. The total volume of the assay was 200 µL and the temperature was set at 37 °C. Readings of the EO/EGCG samples (2.0 mg/mL) and Trolox^®^ working standard solutions were taken using the excitation wavelength set at 485 nm and the emission wavelength at 530 nm for 2 h at 1 min reading interval. After analysis, the data points of the blank, EO sample, EGCG sample, and Trolox^®^ working standards were summed up over time to obtain the area under the fluorescence decay curve (AUC). The ORAC values were calculated using the linear regression (R^2^ = 0.9861) equation (Y = aX + c) between Trolox^®^ concentration (Y) (μM) and the net area (blank-corrected) under the fluorescence decay curve (X). The results were expressed as the mean (±SD) of triplicate measurements in μmol of Trolox^®^ equivalents per liter of the sample tested (μmol TE/L).

#### 3.5.4. Ferric Reducing Antioxidant Power (FRAP) Assay

The FRAP assay was conducted as recommended by Benzie and Strain [68] with slight adjustments. Firstly, the fresh blue FRAP reagent was achieved by mixing 30 mL of acetate buffer, 3 mL of 2,4,6-tris[2-pyridyl]-s-triazine (TPTZ) (Merck, Cat no. T1253) with 3 mL of FeCl_3_ solution and 6.6 mL of distilled water. Then, an L-ascorbic acid (Sigma-Aldrich^®^, Cat no. A5960) standard series of 50 μM, 100 μM, 200 μM, 500 μM, and 1000 μM was prepared from a 1 mM of L-ascorbic acid stock in distilled water. Lastly, in a clear 96-well plate, 300 μL of the FRAP reagent was added to 10 μL of L-ascorbic acid working standard solutions and EO sample (2.0 mg/mL) in triplicate (n = 3). Gallic acid was used as a positive control. For the blank, the phosphate buffer (pH 3.6) was added instead of the sample. The total volume of the assay was 310 μL. The absorbance of TPTZ-Fe (II) in the samples was read at 593 nm at 37 °C for 30 min. The results were calculated using the linear regression (R^2^ = 0.9965) of the L-ascorbic acid (AA) standard series concentrations (μM) and absorbance signals expressed as mean (±SD) of triplicate measurements in μmol L-ascorbic acid equivalents per liter of the sample tested (μmol AAE/L).

### 3.6. Antityrosinase Assay

#### 3.6.1. Essential Oils Samples and Positive Control Preparation

A total of 10 mg/mL of EO working solution was prepared with a DMSO: Tween^®^20 (1:1) solution to facilitate dispersion of the oils then further diluted to 1 mg/mL working solutions with methanol. A 10 mg/mL kojic acid working solution was made up with 100% DMSO and then diluted to 1 mg/mL with methanol.

#### 3.6.2. Tyrosinase Inhibition Assay

The tyrosinase inhibition assay was performed as described previously by Popoola et al. [69] and Cui et al. [70] with slight modifications. The concentrations of EO sample and kojic acid chosen, 200 μg/mL and 50 μg/mL, were achieved by setting up the 96-well plate in the following order: 70 μL of the sample (1 mg/mL) then 30 μL of tyrosinase enzyme (500 U/mL). Each concentration of the sample and positive control was set up in two different wells, whereby one of the wells received enzyme and the other well had no enzyme volume added. All volume deficits were compensated by adding excess buffer. The negative controls, 10% *v*/*v* of 1:1 DMSO: Tween^®^20 in methanol for the EO and 10% *v*/*v* DMSO in methanol for kojic acid were treated the same way. Then, the plate was incubated at 37 °C (±2.0 °C) for 5 min. Thereafter, the reaction was initiated by adding 110 μL of L-tyrosine (2 mM) and subsequently incubated at 37 °C (±2.0 °C) for 30 min. The absorbance of L-DOPA was read at 490 nm on a Multiskan™ spectrum plate reader (Thermo Fisher Scientific, Waltham, MA, USA). Two independent experiments were carried out in triplicate and the percentage tyrosinase inhibition was calculated using Equation (2).

(2) $$Tyrosinase\;inhibition\;\left(\%\right)=\frac{\left(A-B\right)-\left(C-D\right)}{\left(A-B\right)}\times 100,$$

where *A* is the negative control with an enzyme, *B* is the negative control without enzyme, *C* is the EO sample or kojic acid with enzyme and *D* is the EO sample or kojic acid without enzyme. The inhibition percentages were expressed as the mean (±standard deviation) of duplicate measurements. One-way ANOVA was used to compare the absorbance values of the two groups (*p* < 0.05).

### 3.7. Sun Protection Factor (SPF)

The protocol used for this assay was conducted as per Kaur and Saraf [71]. The solubility of the EO in different ratios of ethanol and water was tested by taking 10% to 50% of ethanol in distilled water. The maximum solubility was detected at ethanol: water in a 40:60 ratio above which turbidity developed. Thereafter, an initial stock solution of 1% *v*/*v* was prepared by making up 10 µL of the EO to 1 mL of ethanol: water (40:60). Then out of this stock, 0.1% *v*/*v* was prepared. Subsequently, 100 µL of the EO aliquot and the blank (ethanol: water, 40:60) were injected in the 96-well plate and read in triplicate (n = 3) over the 290–320 nm range at 5 nm interval. The SPF value of the essential oil was calculated following the method by Mansur et al. [43]. The mean of the observed absorbance values was multiplied by their respective erythemogenic effect times solar intensity at wavelength *λ* values, *EE* (*λ*) × *I* (*λ*), then their summation was obtained and multiplied with the correction factor (=10). The calculation is described as Equation (3).

(3) $${\mathrm{SPF}}_{\mathrm{spectrophotometric}}=CF\times \sum_{290}^{320}EE\;\left(\lambda \right)\times I\;\left(\lambda \right)\times Abs\;\left(\lambda \right),$$

where *CF* is the correction factor (=10), *EE* (*λ*) is the erythemogenic effect of radiation at wavelength *λ*, *I* (*λ*) is the solar intensity at wavelength *λ,* and *Abs (λ*) represents the spectrometric absorbance value at wavelength *λ*. The values of *EE* (*λ*) × *I* (*λ*) are constant values that were determined by Sayre et al. [44] as shown in Table 6.

## 4. Conclusions

The present work is the first report to investigate the chemical composition of *O. suffruticosum* essential oil and its biological activities to explore its cosmeceutical potential in selected biological activities of dermatological relevance. The GC-MS analysis served to identify sixteen constituents (**1**–**8**, **11**–**15**, **17**, **18**, **20**) totaling 85.09% of the composition. The monoterpenoids predominated the chemical composition of the essential oil by 84.64%. The major compounds were found to be ketone and alcohol monoterpenes, camphor (**12**) 31.21%, filifolone (**8**) 13.98%, chrysanthenone (**11**) 8.72%, 1,8-cineole (**6**) 7.85%, and terpinen-4-ol (**14**) 7.39%. According to the in vitro biological evaluations conducted, *O. suffruticosum* essential oil possessed low tyrosinase inhibitory activity, low to moderate antibacterial and antioxidant activity, but a promising sun protection ability as per the calculated SPF value. It is further proposed that the therapeutic properties of this essential oil can be improved by the application of nanotechnologies such as nanoencapsulation and nanostructured lipid carriers. This study establishes that the *O. suffruticosum* essential oil can be used as a complementary ingredient to boost the performance of cosmeceuticals with a prominent potential to be used in sunscreen formulations.

## Figures and Tables

**Table 1:** GC-MS analysis of *O. suffruticosum* essential oil.

RT (Min)	Component Code	Mass Spectral Matching	Composition (%)	Experimental RI	Literature RI	Identification
9.214	**1**	α-Pinene	0.80	935	939 ^A^	RI, MS
9.981	**2**	Camphene	2.17	950	950 ^B^	RI, MS
11.374	**3**	Sabinene	0.54	974	973 ^B^	RI, MS
13.928	**4**	α-Terpinene	0.71	1016	1017 ^B^	RI, MS
14.508	**5**	*p*-Cymene	2.45	1026	1024 ^B^	RI, MS
**15.016**	**6**	**1,8-Cineole**	**7.85**	**1035**	**1032** ^B^	**RI, MS**
16.710	**7**	γ-Terpinene	1.48	1061	1060 ^B^	RI, MS
**20.058**	**8**	**Filifolone**	**13.98**	**1109**	**1109** ^Wb^	**RI**
20.372	**9**	Unknown	2.56	1114	-	-
20.560	**10**	Unknown	2.03	1117	-	-
**21.426**	**11**	**Chrysanthenone**	**8.72**	**1131**	**1125** ^B^	**RI, MS**
**23.039**	**12**	**Camphor**	**31.21**	**1155**	**1156** ^Wb^	**RI, MS**
23.683	**13**	Pinocarvone	0.29	1164	1164 ^A^	RI, MS
**25.032**	**14**	**Terpinen-4-ol**	**7.39**	**1183**	**1177** ^B^	**RI, MS**
26.745	**15**	Verbenone	0.56	1207	1206 ^B^	RI, MS
29.015	**16**	Unknown	1.10	1243	-	-
35.372	**17**	Piperitenone	0.78	1339	1341 ^B^	RI, MS
39.371	**18**	3,5-Heptadienal, 2-ethylidene-6-methyl-	5.71	1400	1395 ^Wb^	RI
40.828	**19**	Unknown	3.75	1425	-	-
49.798	**20**	Caryophyllene oxide	0.45	1576	1580 ^B^	RI, MS
Monoterpene hydrocarbons:			8.15			
Oxygenated monoterpenes:			76.49			
Total monoterpenoids:			84.64			
Sesquiterpene hydrocarbons:			0.00			
Oxygenated sesquiterpenes:			0.45			
Total sesquiterpenoids:			0.45			
Total identified:			85.09			
Unidentified:			9.44			
		Total	94.53			

^A^ = Adams [23], ^B^ = Babushok et al. [24], ^Wb^ = NIST Chemistry WebBook [25], MS = In addition to RI, the MS of the analyzed compound matched with the MS of the compound in [23] and/or NIST Chemistry WebBook [25], Unknown = The MS of the compound could not be matched with the available literature data.

**Table 2:** MICs (mg/mL) of *O. suffruticosum* EO and control.

Sample	Micro-Organisms		
	*S. aureus*	*E. coli*	*P. aeruginosa*
*O. suffruticosum*	12.8	12.8	6.4
Ampicillin	<0.2	<0.2	R *

* R = resistant.

**Table 3:** Antioxidant capacities of *O. suffruticosum* essential oil in the DPPH, ABTS, FRAP, and ORAC assays.

Sample		DPPH *	ABTS *			FRAP *	ORAC *
	mg/mL	% RSA6 min ± SD	% RSA6 min ± SD	TEAC (μmol TE/L ± SD)	mg/mL	FRAP (μmol AAE/L ± SD)	ORAC (μmol TE/L ± SD)
*O. suffruticosum*	2	10.03 ± 1.02	87.17 ± 0.76	9431.2 ± 81.5	2	−505.8 ± 80.8	6701.8 ± 57.2
	1	8.38 ± 0.24	81.13 ± 0.51	8784.6 ± 54.5			
	0.5	7.06 ± 0.20	71.46 ± 0.04	7750.1 ± 4.5			
Trolox^®^	2	94.94 ± 0.02	–	–	–	–	–
	1	94.78 ± 0.06					
	0.5	94.45 ± 0.04					
Gallic acid	2	–	97.97 ± 0.13	605,840 ± 27,811.3	2	635,500 ± 4070.9	–
	1		97.96 ± 0.16	355,740 ± 7127.6			
	0.5		98.05 ± 0.03	195,220 ± 6241.5			
EGCG **	–	–	–	–	2	–	26,904 ± 328.2

* Average values of triplicate measurements (n = 3); RSA: radical scavenging activity; SD = standard deviation; RSD = relative standard deviation; TE: Trolox^®^ equivalent; AAE: ascorbic acid equivalent. ** EGCG: (-)-epigallocatechin gallate.

**Table 4:** Summary of the tyrosinase inhibition assay results of *O. suffruticosum* EO at 200 μg/mL and 50.

	Tyrosinase Inhibition (%)	
Samples	at 200 μg/mL	at 50 μg/mL
*O. suffruticosum*	61.46 ± 11.0	26.14 ± 3.74
Kojic acid	96.24 ± 3.62	98.34 ± 0.80

**Table 5:** Spectrophotometric absorbances of hydroalcoholic aliquots of *O. suffruticosum* essential oil and its calculated SPF.

Wavelength (nm)	EE(λ) x I(λ) ** Employed	Absorbance *
290	0.0150	0.2844 ± 0.0075
295	0.0817	0.2759 ± 0.0023
300	0.2874	0.2647 ± 0.0065
305	0.3278	0.2340 ± 0.0053
310	0.1864	0.1919 ± 0.0049
315	0.0837	0.1501 ± 0.0038
320	0.0180	0.1115 ± 0.0030
Calculated SPF		2.299

* Values represent mean absorbance values ± standard deviation of triplicate measurements, n = 3; ** constant values erythemogenic effect (EE) of radiation with wavelength λ x solar intensity (I) at wavelength λ determined by Sayre et al. [44].

**Table 6:** Relationship between erythemogenic effect and radiation intensity.

Wavelength (nm)	EE X I (Normalized)
290	0.0150
295	0.0817
300	0.2874
305	0.3278
310	0.1864
315	0.0837
320	0.0180
Total	1
